# Clinical variability and outcome of succinyl‐CoA:3‐ketoacid CoA transferase deficiency caused by a single OXCT1 mutation: Report of 17 cases

**DOI:** 10.1002/jmd2.12248

**Published:** 2021-09-14

**Authors:** Malak A. Alghamdi, Mohammed Tohary, Hamad Alzaidan, Faiqa Imtiaz, Zuhair N. Al‐Hassnan

**Affiliations:** ^1^ Medical Genetic Division, Pediatrics Department, College of Medicine King Saud University Riyadh Saudi Arabia; ^2^ Department of Medical Genetics King Faisal Specialist Hospital and Research Centre Riyadh Saudi Arabia; ^3^ Department of Clinical Genomics Center of Genomic Medicine, King Faisal Specialist Hospital and Research Centre Riyadh Saudi Arabia

**Keywords:** ketolysis, *OXCT1*, succinyl‐CoA:3‐ketoacid CoA transferase (SCOT) deficiency

## Abstract

Succinyl‐CoA:3‐ketoacid CoA transferase (SCOT) deficiency is an inherited metabolic disease caused by mutated OXCT1 gene resulting in recurrent ketoacidosis. Analysis of longitudinal data in such an ultra‐rare disease is warranted to delineate genotype–phenotype correlations and management outcome. A retrospective analysis of 17 patients, from nine unrelated families, with SCOT deficiency who were followed up in the Medical Genetics Clinic at King Faisal Specialist Hospital and Research Centre was conducted. All the patients were homozygous for p.R468C in *OXCT1* gene. Most of the patients (n = 15, 88.2%) were symptomatic presenting with recurrent ketoacidosis, the onset of which ranged from 6 months to 4 years (median 2 years). A striking inter‐ and intrafamilial variability that ranged from being entirely asymptomatic to death during the first episode. All patients were instructed to avoid fasting, restrict protein in diet, and receive carnitine supplementation. However, there was no correlation between following instructions of chronic management and outcome. Most of the patients had their crises resolved and all of them had normal neurodevelopmental outcome. Our data suggest that SCOT deficiency caused by homozygous p.R468C has variable clinical presentation and incomplete penetrance. The apparent lack of correlation between protein restriction +/− carnitine supplementation and outcome suggests that chronic dietary restriction may not be warranted. However, a longer follow‐up on larger and heterogenous cohort of cases is needed before a clear conclusion on the long‐term management can be reached.

**Synopsis**

Succinyl‐CoA:3‐ketoacid CoA transferase (SCOT) deficiency caused by homozygous p.R468C may have incomplete penetrance. Chronic dietary restriction and carnitine may not be warranted beyond childhood.

## 1 INTRODUCTION

Succinyl‐CoA:3‐oxoacid CoA transferase (SCOT) is the rate‐determining step of ketolysis which takes place in mitochondria of extrahepatic tissues particularly heart, kidney, and brain. First described in 1972,^1^ SCOT deficiency is an autosomal recessive disorder that leads to a ketolytic defect in which extrahepatic tissues cannot utilize the ketone bodies produced by the liver especially during stress resulting in recurrent acute ketoacidosis. Between the ketoacidotic episodes, affected patients are usually asymptomatic; however, persistent ketonemia and ketonuria are observed even in non‐fasting state.^2^ Infancy is the period of highest risk for decompensation which is potentially fatal if not detected early and treated promptly. The long‐term outcome is favorable with normal psychomotor development in most patients.^3^ The mainstay of treatment is suppression of ketogenesis by avoidance of fasting and administration of carbohydrate. Protein‐restricted diet and carnitine supplementation have been suggested as adjunct treatment,^4^, ^5^ and has been practiced in some metabolic clinics.^3^ Sodium bicarbonate supplementation can also be considered in case of persistent metabolic acidosis.

At least 40 *OXCT1* mutations have been reported in patients with SCOT deficiency, most of which are missense.^3^, ^6^ The prevalence of SCOT deficiency is unknown; however, more than 40 cases have been reported in literature. In Saudi Arabia, where consanguinity is high, enrichment of homozygous alleles and preponderance of single founder mutations in recessively inherited diseases are expected. Genetic homogeneity in the presence of a single pathogenic allele provides an opportunity to study the clinical variability and outcome of recessive conditions. In this work, we report the phenotype and long‐term outcome of 17 Saudi patients with SCOT deficiency who were all homozygous for a single *OXCT1* mutation (p. R468C). The implications of dietary restriction and carnitine supplementation on the outcome are also discussed.

## 2 METHODS

A retrospective chart review of 17 patients with SCOT deficiency was conducted. All patients were diagnosed and followed up at King Faisal Special Hospital and Research Centre, Riyadh. The clinical presentation, family history, and confirmatory biochemical and molecular tests were reviewed and recorded. The details of the management including type of dietary restriction, carnitine and bicarbonate supplementation, and compliance to instruction were reviewed as well. The analysis included documented episodes of ketoacidotic crisis and the outcome. The study was approved by the Research Advisory Council of King Faisal Specialist Hospital & Research Centre (RAC #2141 112).

## 3 RESULTS

### 3.1 Clinical phenotype and outcome

The medical records of 17 patients, from nine unrelated consanguineous families, were reviewed. The median follow‐up of the cases was 11 years (range of 5–29 years). Most of the patients (n = 15, 88.2%) were symptomatic presenting with recurrent ketoacidosis. The onset of the first ketoacidotic episode ranged from 6 months to 4 years (median 2 years) with only 3 (20%) patients presenting within the first year.

Two patients were asymptomatic with striking intrafamilial variability. The first patient (case #5, Table 1) was an 8‐year‐old girl who had never experienced a metabolic decompensation. However, one of her affected siblings died during her first episode at the age of 3 years, while her two older sisters, who were 15 and 13 years old respectively, had experienced mild metabolic crises that resolved (family #3, Table 1). The second asymptomatic patient (case #13) was a 19‐year‐old girl who had three affected siblings with history of infrequent episodes of crisis that resolved (family #7, Table 1).

**TABLE 1:** The clinical findings, management, and outcome of 17 patients with SCOT deficiency

Family #	Pt.	Age of onset	Frequency of ketoacidosis crisis	Treatment			Outcome			
				Restricted diet	Carnitine	Sodium bicarbonate	Alive/dead (age)	Ketoacidosis crisis (age of last crisis)	Neurocognitive status	Physical growth
1	1	2 years	3 crises during the second year of life then 2/years	Yes	Yes	Yes	Alive (22 years)	Twice/year	Normal cognition, depression	Underweight (BMI: 16.7)
	2	2 years	2 crises/year	Yes	Yes	Yes	Alive (31 years)	Resolved (8 years)	Normal	Underweight (BMI: 17.9)
2	3	1.5 years	Once/year	No	No	No	Alive (12 years)	Resolved (6 years)	Normal	Normal
3	4	2.5 years	Single episode at the age of 2.5 years	No	No	No	Alive (13 years)	Resolved (2.5 years)	Normal	Normal
	5	–	Asymptomatic	No	No	No	Alive (8 years)	Never	Normal	Normal
	6	3 years	Died during the first crisis	–	–	–	Dead (3 years)	–	Normal	Normal
	7	4 years	Once/year	No	No	No	Alive (15 years)	Resolved (10 years)	Normal	Normal
4	8	6 months	2–3 times/year	Yes	No	Yes	Alive (10 years)	Resolved (8 years)	Normal	Normal
	9	2.5 years	Died during the first crisis	–	–	–	Dead (2.5 years)	–	Normal	Normal
5	10	2 years	3 crises/year	Yes	No	No	Alive (15 years)	Resolved (13 years)	Normal	Normal
6	11	9 months	1 crisis/year	No	Yes	No	Alive (12 years)	Resolved (6 years)	Normal	Normal
7	12	2 years	2 crises/year	No	No	No	Alive (13 years)	Resolved (8 years)	Normal	Normal
	13	–	Asymptomatic	No	No	No	Alive (19 years)	Never	Normal	Normal
	14	2 years	2 crises in life	No	No	No	Alive (21 years)	Resolved (6 years)	Normal	Normal
	15	2 years	Once in life	No	No	No	Alive (24 years)	Resolved (2 years)	Normal	Normal
8	16	8 months	1–2 crises/year	Yes	Yes	No	Alive (9 years)	Resolved (5 years)	Normal	Normal
9	17	2 years	Once/year	Yes	Yes	Yes	Alive (8 years)	Once/year	Normal	Underweight (wt: −2.5 SD)

Abbreviations: BMI, body mass index; SD, standard deviation; wt, weight.

Of the 17 patients, 2 (11.8%) children died during their first episode of ketoacidosis (cases #6 and 9, Table 1). Both cases had severe unresponsive metabolic acidosis that resulted in multi‐organ failure in one child (case #6). Intrafamilial variability was also observed. Case #6 belonged to family #3 where one of the affected children had never experienced a crisis, and the 10‐year‐old sibling of case #9 presented for the first time at the age of 6 months and had been asymptomatic on restricted diet and bicarbonate supplementation.

All patients were instructed to avoid fasting, restrict protein in diet, and receive carnitine supplementation. Sodium bicarbonate supplementation was needed in six patients due to persistent metabolic acidosis. Preventive measures and sick‐day management protocols were provided to all patients after the diagnosis to avoid metabolic crisis. However, not all patients were compliant (Table 1). Only four patients continued to be on both diet restriction and carnitine supplementation, two patients on diet restriction alone, and one patient on carnitine alone. Of these seven cases, metabolic crisis resolved in five. On the other hand, eight patients were not compliant to instructions and had been on normal diet without carnitine supplementation. Metabolic crisis resolved in six while two had never experienced a crisis. In total, out of the 15 symptomatic cases, metabolic crisis resolved in 11 (73.3%) patients whose ages were 9–31 years (median 14 years). The age of the last crisis ranged from 2 to 13 years (median 6 years). In parallel to the resolution of crisis, fasting tolerance also improved with age; adult patients tolerated prolonged fasting (more than 12 hours) with no apparent decompensation.

With the exception of 3 (17.6%) cases, all patients had normal growth, and none of the patient suffered from neurodevelopmental delay. Of note, none of the parents were symptomatic.

Two affected sisters (cases #1 and #2, Table 1) had pregnancy. Pregnancy in the first case was complicated by recurrent ketoacidosis with severe nausea and vomiting requiring several hospital admissions.^7^ Interestingly, pregnancy in her sister (case #2, Table 1) had very mild course with no crisis or requirement of hospitalization. Both sisters had normal delivery of live born infants and had uneventful postpartum period.

### 3.2 Biochemical analysis

All patients had unremarkable plasma acylcarnitine profiles. Persistent ketonuria was not observed in any patient. However, there was no documentation in five cases. The biochemical profiles (pH, CO2, anion gap, electrolytes) during the crises were consistent with what has been previously reported in SCOT deficiency. ^2^, ^3^ Enzyme assay for succinyl CoA‐3‐keto transferase was performed in cultured fibroblasts (by the Biochemical Genetics Laboratory, Oregon Health Sciences University) in one patient (case #11), which showed a level of 1.2 nmol/min/mg protein (normal 2.6–8.6) which was consistent with SCOT deficiency.

### 3.3 Molecular finding

Targeted Sanger sequencing of *OXCT1* confirmed the presence of a homozygous mutation (c1402C>T;p.R468C) in all of the 17 patients. This mutation, which has been previously reported,^2^ was detected in heterozygous state in all of the parents.

## 4 DISCUSSION

In this work, we present the clinical data and outcome of 17 Saudi patients with SCOT deficiency who shared the same homozygous mutation. Most of the cases were symptomatic with episodic metabolic decompensation. However, in spite of the allelic homogeneity, a striking inter‐ and intrafamilial variability was observed. Even within the same family, the variability ranged from death during the first episode, as result of severe unresponsive metabolic acidosis, to an asymptomatic course for the entire life. The variability was also observed during pregnancy in two sisters; one of whom required frequent and prolonged hospitalizations to manage the metabolic derangements, while the other had a very smooth antenatal course.

The natural course of the disease for most of the patients in this cohort followed an episodic metabolic decompensation the frequency of which tended to decrease with age. Our long‐term follow‐up revealed that homozygosity of p.R468C was associated with the highest risk of death and severe episodes during early childhood. Once this critical period was over, the overall outcome was favorable with most children (n = 11, 73.3%) outgrowing the episodic crisis. Of the 15 symptomatic cases, the median age of the last crisis was 6 years, and with the exception of the single case that had crises during pregnancy, none of the others was symptomatic after the age of 13 years.

It is noteworthy that none of the recently reported 44 cases had remained asymptomatic.^3^ However, in our cohort, two of the cases (one child and one adult) had never experienced a metabolic crisis; an observation that highlights the striking clinical variability associated with homozygosity of the p.R468C. Yet, a longer follow‐up would still be needed to assess if they will develop symptoms in older age.

With respect to the management, our data indicate that there was a lack of good correlation between chronic interventions (diet restriction and/or carnitine supplementation) and the phenotypic presentations and outcome. Of the eight patients who were not on diet restriction or carnitine supplementation, metabolic crisis resolved in six and two had never experienced a crisis. And similar to what has been recently reported,^3^ all of our patients had normal neurodevelopmental outcome. Our findings suggest that the chronic management of SCOT deficiency with diet restriction and/or carnitine supplementation may not have an impact on the long‐term outcome, and therefore it may not be needed in adulthood.

Although the cases in our cohort were from 10 unrelated families, all of them had the same mutation (p.R468C) which was previously reported is a patient from another Arab country, Tunisia. This observation suggests that this particular variant is an ancient Arab founder mutation, and therefore it is likely that SCOT cases in other Arab countries may also harbor the same mutation. Hence, it would be prudent to perform a targeted mutation analysis should a case of Arab origin is suspected to have SCOT deficiency. However, testing the entire coding region of OXCT1 may also be performed since other mutations are known to cause SCOT deficiency in Arabs. A more comprehensive molecular analysis, with either multigene panel or whole exome sequencing, may also be considered in cases where the phenotype is not fully explained by SCOT deficiency.

The p.R468C, which does not affect the catalytic activity of succinyl CoA‐3‐keto transferase as it resides far from the active site, was shown to be associated with detectable enzyme activity that was temperature sensitive. The enzyme activity was 12% of the wild‐type value when expressed at 37°C but was much higher at 51% of wild‐type value in the expression at 30°C.^2^ The p.R468C abolishes the salt bridge with Glu312, weakening the network of salt bridges at the core of the C‐terminal domain.^3^

SCOT deficiency had been frequently misdiagnosed in emergency situations as severe gastroenteritis with severe dehydration that manifests as metabolic keto‐acidosis, and subsequently treated as so with complete recovery without further investigation. However, delayed diagnosis of SCOT deficiency might result in drastic consequences as death could be a result of the first decompensation. Early diagnosis and prompt management of crisis are expected to lead to favorable outcome. Contrary to what has been reported in p.R468C homozygotes,^2^ our cases showed lack of permanent ketonuria; a note that was also observed in the cohort reported by Grünert et al.,^3^ which warrants the attention of clinicians when they evaluate the differential diagnosis of recurrent metabolic crisis of unclear etiology.

## 5 CONCLUSION

Our data suggests that SCOT deficiency caused by homozygous p.R468C is a potentially fatal disorder that has intrafamilial variability with incomplete penetrance. Chronic management with protein restriction and carnitine supplementation may not be warranted. However, prompt treatment of acute decompensation is imperative and should remain a life‐long standard of care with full attention during stresses. Longitudinal studies on larger and heterogenous cohort of cases are still needed before a clear conclusion on the long‐term management can be reached.

## CONFLICT OF INTEREST

Malak A Alghamdi, Mohammed Tohary, Hamad Alzaidan, Faiqa Imtiaz, and Zuhair N Al‐Hassnan have no conflict of interest.

## AUTHOR CONTRIBUTIONS

Conception and design of the study: Zuhair N. Al‐Hassnan; Drafting the manuscript: Malak A. Alghamdi; Data analysis and interpretation: Malak A. Alghamdi, Mohammed Tohary, Faiqa Imtiaz, Hamad Alzaidan, Zuhair N. Al‐Hassnan; Evaluation of manuscript: Zuhair N. Al‐Hassnan.

## ETHICS STATEMENT

The study was approved by the Research Advisory Council (RAC) of King Faisal Specialist Hospital & Research Centre (RAC #2141112). All procedures followed were in accordance with the ethical standards of the RAC on human experimentation and with the Helsinki Declaration of 1975, as revised in 2000. Since the study was a retrospective chart review, written informed consent was waived by the RAC.

## INFORMED CONSENT

A waiver of written informed consent was approved by the Research Advisory Council of King Faisal Specialist Hospital & Research Centre.

## Data Availability

This manuscript has associated data in a data repository, including all data for which data deposition is mandatory.
